# An Integrated Analysis of mRNA and lncRNA Expression Profiles Indicates Their Potential Contribution to Brown Fat Dysfunction With Aging

**DOI:** 10.3389/fendo.2020.00046

**Published:** 2020-02-17

**Authors:** Jie Feng, Haoqin Xu, Fenghui Pan, Jiaojiao Hu, Yulin Wu, Ning Lin, Xiaoxiao Zhang, Chenbo Ji, Yun Hu, Hong Zhong, Linping Yan, Tianying Zhong, Xianwei Cui

**Affiliations:** ^1^Nanjing Maternity and Child Health Care Hospital, Women's Hospital of Nanjing Medical University, Nanjing, China; ^2^Jiangsu Institute of Planned Parenthood Research, Nanjing, China; ^3^Department of Geriatrics, Drum Tower Hospital Affiliated to Nanjing University Medical School, Nanjing, China

**Keywords:** BAT dysfunction, thermogenesis, RNA-sequencing, GSEA analysis, Pparα

## Abstract

Brown adipose tissue (BAT) can convert fatty acids and glucose into heat, exhibiting the potential to combat obesity and diabetes. The mass and activity of BAT gradually diminishes with aging. As a newly found regulator of gene expression, long non-coding RNAs (lncRNAs) exhibit a wide range of functions in life processes. However, whether long non-coding RNA (lncRNA) involves in BAT dysfunction with aging is still unclear. Here, using RNA-sequencing technology, we identified 3237 messenger RNAs (mRNAs) and 1312 lncRNAs as differentially expressed in BAT of 10-months-old mice compared with 6- to 8-week-old. The protein-protein interaction network and k-score analysis revealed that the core mRNAs were associated with two important aging-related pathways, including cell cycle and p53 signaling pathway. Gene set enrichment analysis indicated that these mRNAs might participate in lipid metabolism and brown fat dysfunction. Functional enrichment analyses demonstrated that dysregulated lncRNAs were associated with mitochondria, regulation of cellular senescence, cell cycle, metabolic and p53 signaling pathways. Moreover, we revealed that two lncRNAs (NONMMUT024512 and n281160) may involve in the regulation of their adjacent gene peroxisome proliferator-activated receptor alpha (Pparα), a thermogenesis regulator. Collectively, these results lay a foundation for extensive studies on the role of lncRNAs in age-related thermogenic degradation.

## Introduction

Brown adipose tissue (BAT) is an adaptive thermogenic organ that transforms chemical energy into heat in the presence of cold exposure (1). BAT contains abundant mitochondria and is characterized by the enrichment of unique uncoupling protein 1 (UCP1), which is located within the inner mitochondrial membrane and dissolves the mitochondrial proton gradient for heat generation (2). Although the content of BAT accounts for only a relatively small proportion of the body, 50 g of active BAT can approximately generate 20% of daily energy consumption (3). In addition to being abundant in babies, there is also a certain percentage BAT in adults (4). The present research also demonstrates that the mass of human BAT in adults is inversely proportional to body mass index, obesity and glucose level (5). These findings indicate the critical role of BAT in human metabolism regulation.

The amount and function of human BAT quickly reaches a peak after birth and gradually deteriorates throughout life (6). Particularly in mice, BAT gradually disappears at approximately mid-age. Evidence demonstrates that the involution of brown adipocyte regeneration may be endogenously controlled and is due to age-induced dysfunction of brown adipogenic stem/progenitor cell (7). Recent studies have also suggested that several changes in endocrine signaling molecules, such as sex hormones, inflammatory cytokines, and glucocorticoids, could act a significantly inhibitory effect on brown adipogenesis (7, 8). Furthermore, a number of studies have revealed that Sirtuin 1 (SIRT1) is an important regulator of UCP1 and possibly regulates adipose tissue browning for adaptive thermogenesis (9). The expression of UCP1 can be up-regulated by enhancing SIRT1 activity in mature brown adipocytes (10). However, SIRT1 is reduced with aging (11), and SIRT1 deficiency is associated with decreased thermogenesis and BAT degeneration (12). Previous evidences suggest that SIRT1 regulates the expression and activity of peroxisome proliferator-activated receptor alpha (PPARα) (13), which is highly expressed in BAT (14) and plays a vital role in lipid oxidation. Interestingly, PPARα also has been shown to be associated with aging (15). In several tissues, such as kidney, heart and spleen, PPARα expression or activity is decreased during aging (16–18). Furthermore, PPARα deficiency accelerates renal aging and impairs lipid metabolism in the kidney (19). However, it is unclear whether PPARα involves in BAT dysfunction with aging. Although several aspects are tightly associated with less functional BAT, the predisposition mechanism hasn't been identified, and the molecular reasons for age-related thermogenic dysfunction have largely remained unaddressed.

Accumulating evidence suggests that long non-coding RNAs (lncRNAs), transcripts longer than 200 nt without distinct protein coding capacity (20), are implicated in the regulatory network of brown adipogenesis and thermogenesis. This type of regulatory capacity has been exemplified by a set of BAT-specific lncRNAs, such as Blnc1 and lnc-BATE1. Blnc1 forms a feed forward control network with Early B Cell Factor 2 to drive adipogenesis toward a thermogenic phenotype (21). Adopting silencing and rescuing strategies, another study proposed that lnc-BATE1 is closely associated with BAT character and thermogenic capacity (22). As an age-related lncRNA, uc.417 is down-regulated in response to cold and other thermogenic signals in BAT and attenuates BAT activity via a p38MAPK signaling pathway-dependent mechanism (23). Although emerging evidence has demonstrated roles for lncRNAs in the adipogenesis and thermogenesis of BAT, it is still unclear whether lncRNAs contribute to diminished BAT function with aging.

A previous study observed that aging was accompanied by morphologic abnormalities and thermogenic dysfunction of BAT in mice (24). In old mice vs. to younger mice, BAT is populated by adipocytes having morphologic characteristics of white adipocytes, and UCP1 expression is markedly declined. Here, using RNA-sequencing (RNA-seq) technology, we identified 3,237 messenger RNAs (mRNAs) and 1,312 lncRNAs that were differentially expressed {fold change (FC) ≥ 2.0 and *q*-value [also known as false discovery rate (FDR)] ≤ 0.05} in aged mice (10-month-old). The protein-protein interaction network and k-score analysis revealed that the core mRNAs were associated with cell cycle and p53 signaling pathway. Gene set enrichment analysis (GSEA) indicated that these mRNAs might participate in lipid metabolism and brown fat dysfunction. Moreover, Gene Ontology (GO) and pathway analyses demonstrated that these lncRNAs were involved in cellular response to stress, regulation of cellular senescence and mitochondria, and were linked to cell cycle, metabolic and p53 signaling pathways. Finally, we evaluated the relationship between two lncRNAs (NONMMUT024512 and n281160) and their neighboring coding gene Pparα. Together, our preliminary research lays a foundation for extensive studies on the impact of lncRNAs on BAT with aging, particularly with regard to thermogenic degradation.

## Materials and Methods

### Animal and Tissue Preparation

Male C57BL/6 mice were acquired from the Model Animal Research Center of Nanjing University. The mice were sustained on a 12/12 h light/dark cycle at 23°C. All groups were fed standard chow and had free access to water and food (23). Murine classical brown depots are in the interscapular region. The mice were sacrificed by cervical dislocation at 6–8 weeks and 10 months of age, and the interscapular BAT was carefully separated from the surrounding tissues. Each BAT sample was immediately frozen in liquid nitrogen and then stored at −80°C. Furthermore, all animal experiments were approved by the Nanjing Medical University Committee on Care and Use of Animals, and conducted in conformity with the Guide for the Care and Use of Laboratory Animals published by the U.S. National Institutes of Health (NIH Publication number 85–23, revised 1996).

### Histologic Analysis

BAT tissues from mice were soaked in 4% paraformaldehyde and processed routinely. After fixation for nearly 24 h, the tissues were embedded in paraffin and cut into ~5-mm-thick sections for histology and immunohistochemistry (IHC) (24). Then, these sections were mounted onto glass slides. Multiple slides were stained with Haematoxylin and eosin (H&E) for general morphologic observation. For IHC, the slides were incubated overnight at 4°C with primary antibodies against UCP1 (1:500 dilution) (ab209483; Abcam, Cambridge, United Kingdom). Immunostaining was performed with anti-rabbit horseradish peroxidase-tagged secondary antibodies, and a Diaminobenzidine Detection Kit (EliVision, Incheon, Korea) was applied for color development. Then, haematoxylin was used for counterstaining. All images were acquired using a fluorescence microscope (Imager.A2; Zeiss, Oberkochen, Germany).

### RNA Extraction, Library Construction, and Sequencing Analysis

According to the manufacturer's protocol, total RNA was extracted from frozen BAT samples using the mir Vana^TM^ miRNA Isolation Kit (Ambion, Carlsbad, CA, USA). Then, the Agilent Bioanalyzer 2100 (Agilent Technologies, Santa Clara, CA, USA) was applied to examine the RIN number of total RNA to identify RNA integrity. Eligible total RNA was next purified using the RNAClean XP Kit (Beckman Coulter, Inc. Kraemer Boulevard Brea, CA, USA). In addition, the contamination of genomic DNA was eliminated by using an RNase-Free DNase Set (QIAGEN, GmBH, Germany), and ribosomal RNA was also removed by the Ribo-Zero™ rRNA Removal Kit (Epicenter, Madison, WI, USA). An Illumina TruSeq™ RNA Sample Prep Kit was used to construct RNA-seq libraries, and the Agilent Bioanalyzer 2100 (Agilent Technologies, Santa Clara, CA, USA) was used to identify the quality of all libraries. Furthermore, one lane of a 100 + 100-nt paired-end Illumina HiSeq 2500 was applied to analyse the libraries with Illumina sequencing primers (25). Then, an Illumina HiSeq 2500 platform was applied to perform RNA-seq.

We filtered the low-quality reads from the raw reads with Seqtk software (https://github.com/lh3/seqtk) to obtain clean reads for data analysis. Mapping of all clean reads was carried on the mouse reference genome [GRCm38.p4 (mm10)] (ftp://ftp.ensembl.org/pub/release-83/fasta/mus_musculus/dna/Mus_musculus.GRCm38.dna.primary_assembly.fa.gz) using Hisat2 (version: 2.0.4) (26) with parameters set to default values. Then, we first applied StringTie (version: 1.3.0) (27, 28) to count the fragments of each gene, and further used trimmed mean of M values (TMM) (29) method to perform normalization. Finally, we used the perl script to calculate the Fragments Per Kilobase of exon model per Million mapped reads (FPKM) value of each gene. All high-quality reads in this study have been submitted to NCBI's Sequence Read Archive under accession number SRP150130. The quality control information including correlation between replicates, total number of reads, mapping ratio as well as unique mapped reads ratio was already provided in Figure S1 and Table S1.

In the present study, the filtering criteria of lncRNAs are as follows: 1. Extract category {i, u, x}; 2. length ≥ 200 bp, Exon number ≥ 2; 3. ORF <300 bp. Moreover, the coding potential prediction criteria of lncRNAs are as follows: 1. CPC score <0; 2. CNCI score <0; 3. Pfam not significant.

### Functional Enrichment Analyses

GSEA analysis was performed using the online GSEA software (https://www.broadinstitute.org/gsea/) with default parameters to explore the function of all mRNAs. Similar to previous studies (30), differentially expressed (DE) lncRNAs were chosen to predict target genes, and then the predicted candidate lncRNAs were integrated with DE mRNAs in the profile. GO analysis (http://www.geneontology.org/) was applied to identify the potential functions of DE mRNAs related to DE lncRNAs. Then, a pathway analysis was performed to investigate the significant pathways of DE mRNAs associated with DE lncRNAs, following the Kyoto Encyclopedia of Genes and Genomes (KEGG; http://www.genome.jp/kegg).

### Quantitative Real-Time PCR (qPCR)

BAT samples were minced using a homogenizer (IKA, Staufan, Germany), and total RNA was isolated from these samples with TRIzol reagent (Invitrogen, Carlsbad, CA, USA). The concentration and purity of the extracted RNA were assessed with a NanoDrop 2000 spectrophotometer (Thermo Fisher Scientific, Waltham, MA, USA). Then, complementary DNA was acquired from reverse transcription of 500 ng RNA using the PrimeScript™ RT regent kit (Takara, Mountain View, CA, USA). All primers were designed online with Primer 3 (http://sourceforge.net/projects/primer3/), and the uniqueness of their amplification products was checked using the Basic Local Alignment Search Tool of NCBI. The sequences of these primers are listed in Table S2. The qPCR analyses were performed on the ViiA™ 7 DX Real-Time PCR System (Applied Biosystem) with a SYBR Green Kit (TaKaRa, Tokyo, Japan), and β-actin was used as the internal control.

### Protein Preparation and Western Blot

BAT samples were minced by a homogenizer (IKA, Staufan, Germany), and lysed using RIPA buffer (Beyotime Biotechnology, Shanghai, China) containing protease inhibitor cocktail (1 tablet/10 ml, Roche, CA, USA) at 4°C with gentle shaking. Protein concentrations were detected with the BCA Protein Assay Kit (Thermo Fisher Scientific). Proteins were loaded on a 4–12% SDS-PAGE gel for electrophoresis, transferred to PVDF membranes, and immunoblotted with specific primary antibodies as follows: rabbit monoclonal UCP1 (Ab155117; Abcam, MO, USA; 1:1,000 dilution) and rabbit monoclonal GAPDH (AF1186; Beyotime Biotechnology, Shanghai, China; 1:5,000 dilution). Furthermore, the secondary antibody was horseradish peroxidase-conjugated goat anti-rabbit IgG (BL003A; Biosharp, Hefei, China; 1:5,000 dilution).

### Primary Brown Adipocyte Cultures and Small Interfering RNA Transfection

Brown pre-adipocytes were obtained from interscapular BAT from 4-week-old mice. BAT was isolated, cut into small pieces with scissors and then digested using 0.2% collagenase I (Sigma-Aldrich) at 37°C for 1 h with gentle inversions. The digested solution was filtered through 100 μm nylon meshs (Corning, NY) followed by centrifuging at 1,000 rpm for 10 min. Then, the isolated cells were resuspended in pre-warmed Dulbecco's Modified Eagle Medium (DMEM; Gibco, Carlsbad, CA) contained 10% fetal bovine serum (FBS; Gibco), 1% penicillin/streptomycin (P/S; Gibco), and seeded into 6 well plates.

For knockdown, cells at 50% confluence were transfected with small interfering RNA (siRNA) pool (Table S2) using Lipofectamine 3000 (Thermo Fisher Scientific) at 200 nM in Opti-MEM medium (Thermo Fisher Scientific). After 24 h of infection, the medium was changed. Pre-adipocytes were cultured until achieving post confluency, followed by induction of differentiation. The differentiation process was initiated with a cocktail containing Preadipocyte Differentiation Medium (PADM; Sciencell, Carlsbad, CA), 5% FBS (Sciencell), 1% P/S (Sciencell), and 1% Preadipocyte Differentiation Supplement (PAdDS; Sciencell). Six days after induction, the cells were switched to DMEM supplemented with 5% FBS and 1% P/S. The cells were harvested with Trizol at the indicated time for further analysis.

### Oil Red O Staining

Adipocytes at day 8 of differentiation were washed twice with phosphate-buffered saline (PBS; Gibco, Carlsbad, CA) and subsequently fixed with 4% paraformaldehyde for 30 min. After washing twice with PBS, the fixed cells were stained using 0.2% Oil red O (Sigma-Aldrich, St. Louis, MO) solution for 30 min at 37°C. Then, the stained cells were visualized using an Observer D1 microscope (Carl Zeiss, Werk Gottingen, Germany).

### Cell Immunofluorescent Staining

At day 8 of differentiation, brown adipocytes were washed by PBS twice and fixed using 4% paraformaldehyde at room temperature for 15 min, followed by permeabilized treatment with PBS containing 0.25% Triton X-100 (PBST) for another 20 min. The cells were blocked using 2.5% BSA in PBST for 30 min at 37°C, and then incubated with UCP1 antibody (Ab155117; Abcam, MO, USA; 1:1,000 dilution) overnight at 4°C. Subsequently, the cells were stained with the secondary antibodies conjugated with Alexa Fluor 546 (Invitrogen, 1:1,000 dilution) for 1 h at 37°C in the dark. After the cells were washed by PBS 3 times, nuclei labeling were counterstained with DAPI (Invitrogen, 1:10,000 dilution) for 5 min. At last, the cells were mounted on a microscope slide using Fluoromount-G mounting medium. All images were acquired using a fluorescence microscope (Imager.A2; Zeiss, Oberkochen, Germany).

### Statistical Analysis

The differential analysis of gene expression in RNA-seq data was performed using the edgeR package (31). Only the genes with the criteria of FC ≥ 2 and FDR ≤ 0.05 were identified as differentially expressed. Furthermore, qPCR data were analyzed with GraphPad Prism 7.0 (San Diego, CA, USA), and the results are expressed as the means ± standard deviation. A two-sided unpaired Student's *t*-test was performed to examine the differences of qPCR results in young and old mice.

## Results

### The Function of BAT Is Dramatically Reduced in 10-Month-Old Mice

**Figure 1:**
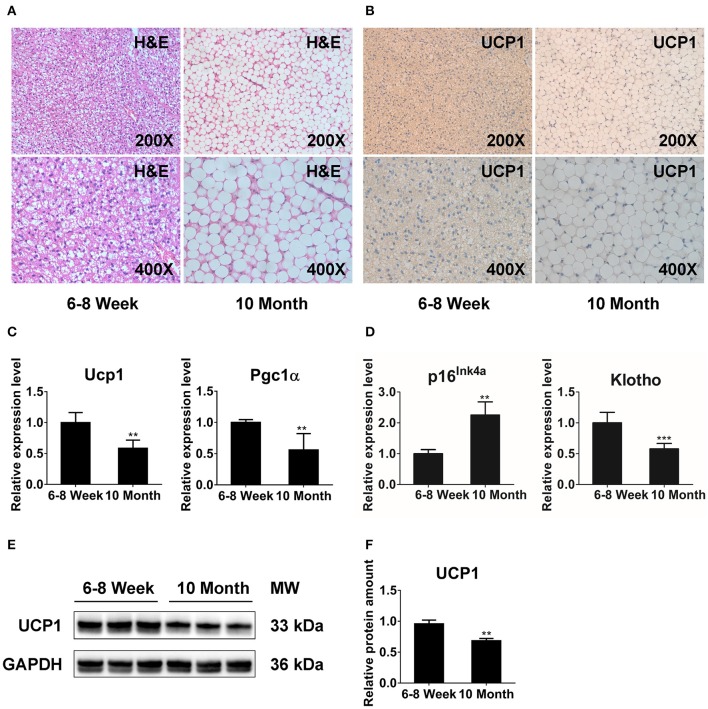
The function of BAT is dramatically reduced in 10-months-old mice. H&E staining **(A)** and immunohistochemistry analysis against UCP1 **(B)** in interscapular BAT from 6- to 8-week-old and 10-months-old mice. **(C,D)** qPCR analysis of the thermogenesis and senescence associated genes in interscapular BAT. **(E,F)** Western blot for UCP1 and GAPDH in BAT of young and old groups. Data are presented as the mean ± standard deviation (S.D.). Statistical significance evaluated by unpaired Student's *t*-test is reported as **p < 0.01 and ***p < 0.001.

We performed histologic analysis to explore the morphology and function of BAT with aging. H&E staining showed that BAT from 6- to 8-week-old mice (young mice) displayed unique multilocular adipocytes (Figure 1A). However, brown adipocytes in 10-month-old mice (old mice) exhibited white-like unilocular formation with numerous lipid droplets (Figure 1A). Furthermore, the results from IHC revealed that UCP1 expression was significantly decreased in old mice relative to that in young mice (Figure 1B). We also conducted qPCR analysis to explore the impact of morphologic abnormalities on thermogenic regulators, including Ucp1 and peroxisome proliferator-activated receptor gamma coactivator 1-alpha (Pgc1α). The expression of Ucp1 and Pgc1α was markedly declined in older animals (Figure 1C). Additionally, we detected the expression of senescence markers (p16^Ink4a^ and Klotho) (32, 33) to determine the senescent status of BAT. Notably, BAT of old mice exhibited markedly increased p16^Ink4a^ expression and a significant reduction of Klotho expression compared with that in the younger group (Figure 1D). The western blot results showed that the protein level of UCP1 was reduced in BAT from old mice (Figures 1E,F). Together, these observations indicate that distinct multilocular brown adipocytes gradually exhibit white-like unilocular morphology. This finding, along with expressional abnormalities of thermogenic markers, suggests that thermogenic capacity may diminish in old mice.

### mRNA Expression Profiling in BAT From 10-Month-Old Mice Compared With 6- to 8-Week-Old Mice

**Figure 2:**
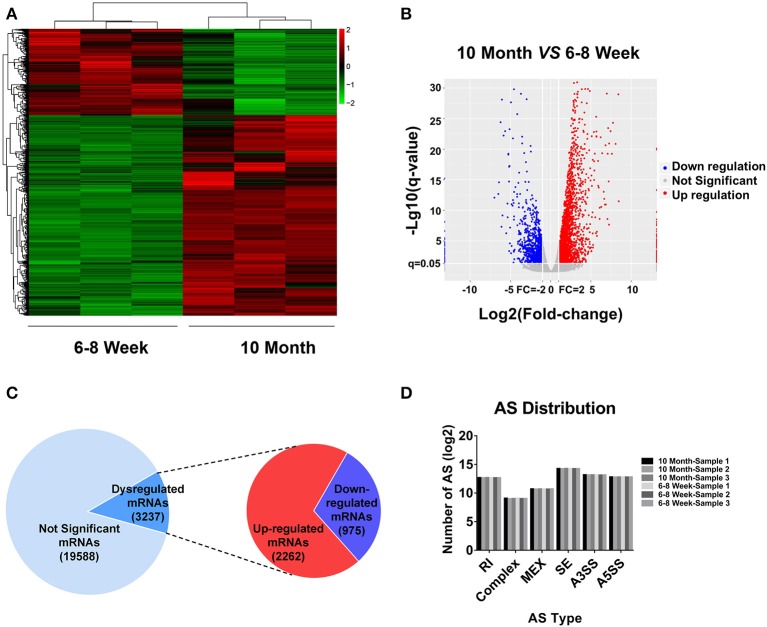
mRNA expression profiling in BAT from 10-month-old mice compared with 6- to 8-week-old mice. **(A)** Clustered heat map analysis of differentially expressed mRNAs. **(B)** The volcano plot was used to evaluate the variation in mRNA expression. **(C)** The total mRNAs detected by RNA-seq and differentially expressed mRNAs between two groups. **(D)** Distribution of different types of alternative splicing (AS) events in six samples. RI, Retained intron; MEX, Mutually exclusive exon; SE, Skipped exon; A3SS, Alternative 3' splicing site; A5SS, Alternative 5′ splicing stie.

According to the RNA-seq data, a heat map and clustering analyses were applied to display the differential expression of mRNAs in old mice relative to the young group (Figure 2A). In addition, the variation of mRNA expression among the two groups was also exhibited by a volcano plot (Figure 2B). Here, we totally detected 22,825 mRNAs in BAT. Of these, 3,237 mRNAs were identified as differentially expression (FC ≥ 2.0 and FDR ≤ 0.05) in aged mice (Figure 2C). 2,262 mRNAs were up-regulated, whereas 975 mRNAs showed a down-regulated pattern. All DE mRNAs were listed in Table S3. Alternative splicing (AS) is an important mechanism for generating protein and functional diversity. In higher eukaryotes, one pre-mRNA can generate multiple mRNA isoforms through AS. There are mainly 7 types of AS in living organisms, as follows: Skipped exon (SE), Alternative 5' splicing stie (A5SS), Alternative 3' splicing site (A3SS), Retained intron (RI), Mutually exclusive exon (MEX), Alternative promoters (AP), and Alternative poly(A) (APA). To investigate the relative importance of AS modes, we applied Astalavista (version: 3.1) (34–36) to detect the alternative splicing sites and conducted statistics analysis on various splicing forms. SE predominated, A3SS was the second most prevalent mode, and complex was the least frequent (Figure 2D). We further used MISO software (37) for quantitative analysis of AS. The positions and expression results of AS in all samples were listed in Table S4.

### Validation of Differentially Expressed mRNAs by qPCR

**Figure 3:**
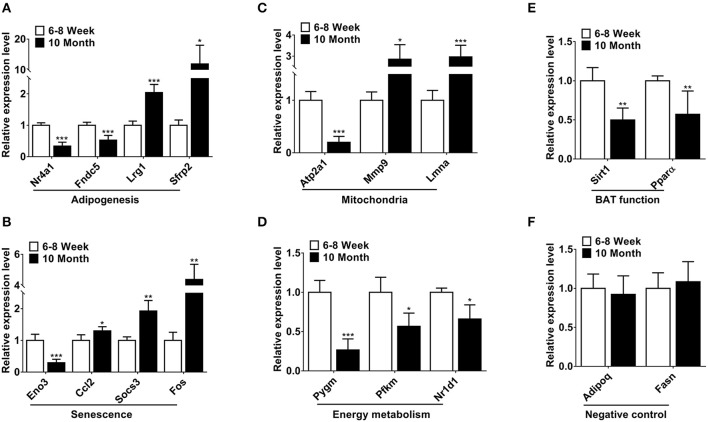
Validation of differentially expressed mRNAs by qPCR. **(A–D)** The differentially expressed mRNAs involved in adipogenesis, senescence, mitochondria and energy metabolism. **(E)** The expression of two BAT function associated genes. **(F)** The negative controls in the qPCR validations. *p < 0.05, **p < 0.01 and ***p < 0.001. (Student's *t-*test, *n* = 6 per group).

Based on relatively high abundance, FC ≥ 2, *p* < 0.001 and FDR < 0.05, we selected 14 mRNAs to validate their expression. The function of these genes was associated with adipogenesis, senescence, mitochondria and energy metabolism. As shown in Figures 3A–D, the qPCR results were consistent with the expression trends of the high-throughput sequencing (Table S3). Furthermore, we detected the expression of two BAT function associated genes. Of these, Sirt1 can regulate adaptive thermogenesis through enhancing Ucp1 expression, while Pparα is a key thermogenic marker of BAT. As expected, the expression of these two genes was declined in old mice (Figure 3E). Two highly-expressed genes Adipoq and Fasn were selected as negative controls for qPCR verification in BAT (Figure 3F).

### lncRNA Expression Profiling in BAT From 10-Month-Old Mice Compared With 6- to 8-Week-Old Mice

**Figure 4:**
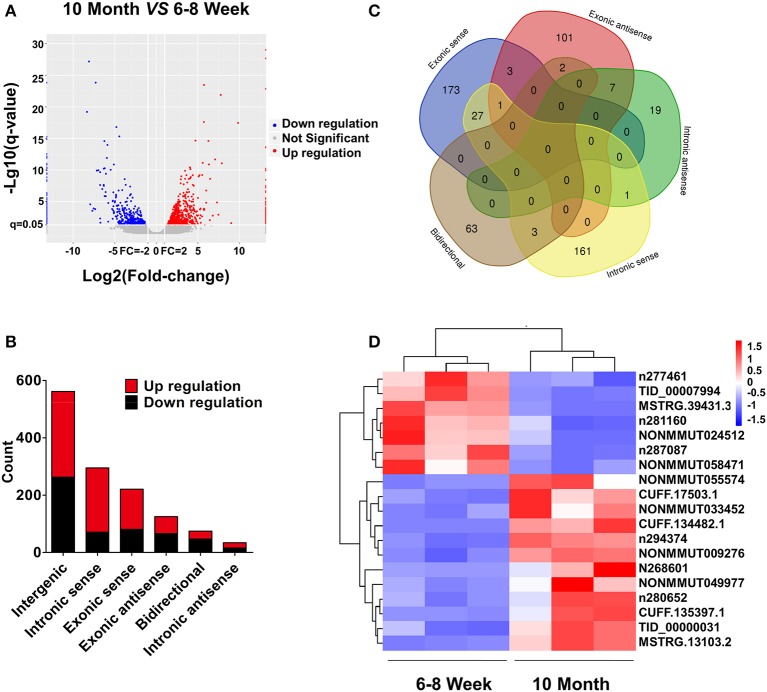
lncRNA expression profiling in BAT from 10-month-old mice compared with 6- to 8-week-old mice. **(A)** The volcano plot is applied to evaluate the variation of lncRNA expression. **(B)** Annotation of genomic context of differentially expressed lncRNAs. **(C)** Venn diagram reveals overlapping relationships of adjacent genes between different categories of lncRNAs. **(D)** Expression pattern of validated lncRNAs.

Based on the RNA-seq data, the clustered heat map analysis was used to display differentially expressed lncRNAs in BAT from two groups (Figure S2). As a visualization method, a volcano plot was applied to show the variation in lncRNA expression between the two groups (Figure 4A). We identified 1,312 DE lncRNAs in young and old mice: 768 up-regulated and 544 down-regulated (FC ≥ 2.0 and FDR ≤ 0.05). According to the position relation of lncRNAs and adjacent mRNAs, these lncRNAs were classified into six categories: 42.8% were intergenic, 5.7% were bidirectional, 9.5% were exonic antisense, 16.8% were exonic sense, 22.5% were intronic sense, and 2.6% were intronic antisense (Figure 4B). There are overlaps among adjacent mRNAs of different categories lncRNAs (Figure 4C). Moreover, we selected a series of lncRNAs to validate their expression. The heat map and clustering analyses were used to reveal the expression patterns of these lncRNAs in different samples (Figure 4D). All DE lncRNAs were listed in Table S5.

### Validation of Differentially Expressed lncRNAs Using qPCR

**Figure 5:**
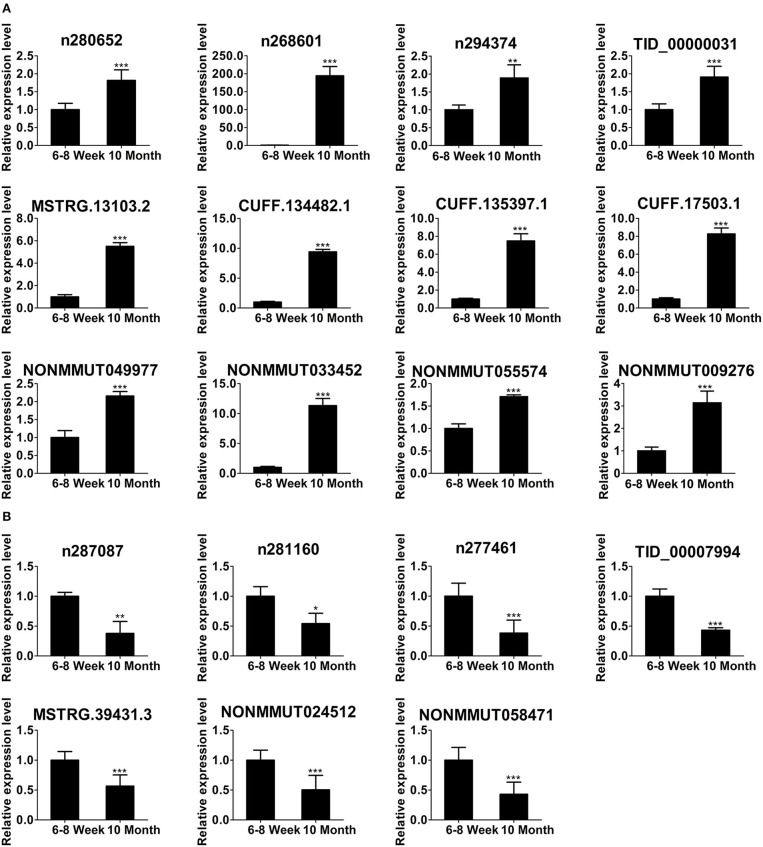
Validation of differentially expressed lncRNAs using qPCR. The up-regulated lncRNAs **(A)** and down-regulated lncRNAs **(B)** in 10-month-old mice compared with 6- to 8-week-old mice. *p < 0.05, **p < 0.01, and ***p < 0.001. (Student's *t-*test, *n* = 6 per group).

To focus our validation efforts, we confirmed some candidate lncRNAs by relatively high abundance, FC ≥ 2, *p* < 0.01, FDR < 0.05 and their adjacent genes. In parallel with the RNA-seq data (Figure 4D), qPCR results showed that the expression of n280652, n268601, n294374, TID_00000031, MSTRG.13103.2, CUFF.134482.1, CUFF.135397.1, CUFF.17503.1, NONMMUT049977, NONMMUT033452, NONMMUT055574, and NONMMUT009276 was increased (Figure 5A). The expression of n287087, n281160, n277461, TID_00007994, MSTRG.39431.3, NONMMUT024512, and NONMMUT058471 was decreased in old mice (Figure 5B). Specifically, using searching Cistrome Data Browser (38–40) and Animal Transcription Factor Database, we found that the transcription sites of two down-regulated lncRNAs, n281160, and NONMMUT024512, positioned in the vicinity of Pparα in nuclei.

### Functional Enrichment Analyses of the mRNAs

**Figure 6:**
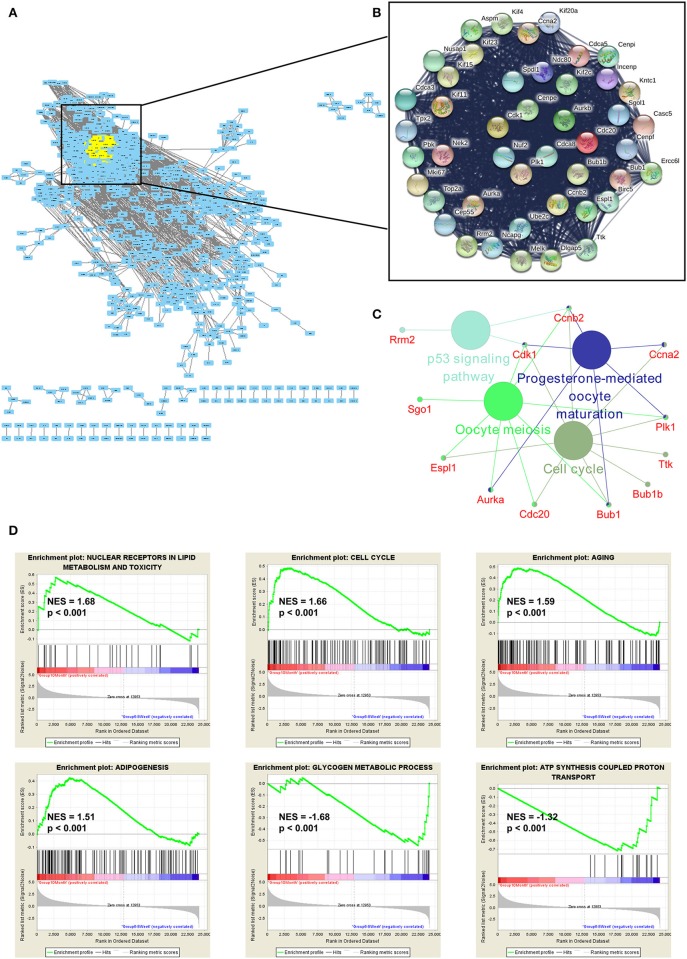
Functional enrichment analyses of the mRNAs. **(A)** The protein-protein interaction (PPI) network was constructed by Search Tool for the Retrieval of Interacting Genes/Proteins. The minimum required interaction score was set as 0.9. **(B)** The k-score analysis was used to assess the core subnetwork of the PPI network. **(C)** The hub genes in this subnetwork were associated with two important aging-related pathways, including cell cycle and p53 signaling pathway. **(D)** GSEA analyses of gene sets for nuclear receptors in lipid metabolism and toxicity, cell cycle, aging, adipogenesis, glycogen metabolic process and ATP synthesis coupled proton transport. NES, normalized enrichment score.

To explore the function of DE genes at the protein level, we adopted Search Tool for the Retrieval of Interacting Genes/Proteins (STRING) to reveal an interaction network for annotating functional properties of proteins (41). Based on the minimum required interaction score (0.9), the network was constructed. There were 1,068 nodes and 3,875 edges in this network (Figure 6A). The k-score analysis was chosen to assess the core subnetworks in this network. After calculating k-score for each subnetwork, we identified a core subnetwork with k-score as 33.909. This subnetwork consisted of 45 nodes and 986 edges (Figure 6B). Since pathway analysis is significant for understanding the functions of these 45 hub genes, we also applied Cytoscape (Version 3.7.1) to visualize pathway information of these genes. The results showed that these genes were associated with two important aging-related pathways, including cell cycle and p53 signaling pathway (Figure 6C). Furthermore, GSEA analysis demonstrated that up-regulated genes were related to nuclear receptors in lipid metabolism and toxicity [normalized enrichment score (NES) = 1.68, *p* < 0.001], cell cycle (NES = 1.66, *p* < 0.001), aging (NES = 1.59, *p* < 0.001), and adipogenesis (NES = 1.51, *p* < 0.001), whereas down-regulated genes were functionally associated with glycogen metabolic process (NES = −1.68, *p* < 0.001) and ATP synthesis coupled proton transport (NES = −1.32, *p* < 0.001; Figure 6D).

### GO and Pathway Analyses of mRNAs Associated With Differentially Expressed lncRNAs

**Figure 7:**
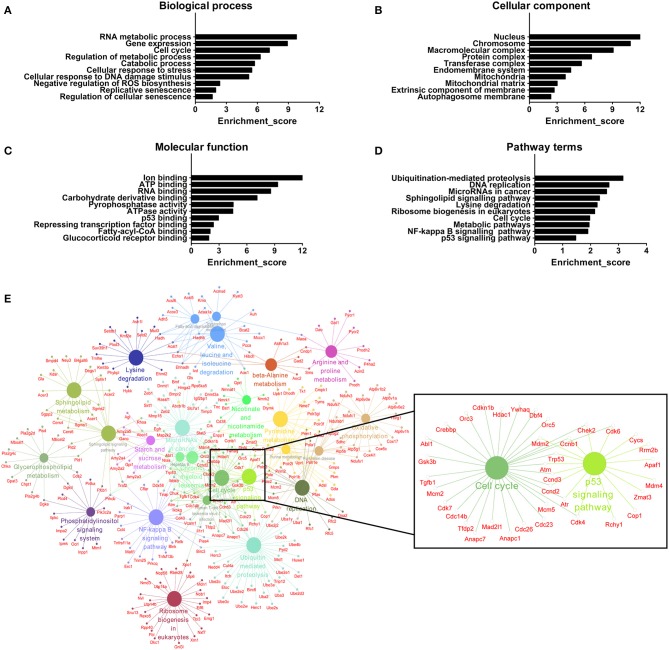
GO and pathway analyses of mRNAs associated with differentially expressed lncRNAs. **(A)** The biological process categories. **(B)** The cellular component categories. **(C)** The molecular function categories. **(D)** Canonical signaling pathways. **(E)** Interaction and overlaps of associated genes among significantly enriched pathways.

We further adopted GO analysis to observe the potential function of mRNAs related to DE lncRNAs. GO analysis identified RNA metabolic process, gene expression, cell cycle, regulation of metabolic process, catabolic process, cellular response to stress, cellular response to DNA damage stimulus, negative regulation of reactive oxygen species (ROS) biosynthesis, replicative senescence and regulation of cellular senescence as biological processes associated with DE lncRNAs (Figure 7A). Regarding cellular components, the most highly enriched terms were nucleus, chromosome, macromolecular complex, protein complex, transferase complex, endomembrane system, mitochondria, mitochondrial matrix, extrinsic component of membrane, and autophagosome membrane (Figure 7B). For molecular functions, ion binding, ATP binding, RNA binding, carbohydrate derivative binding, pyrophosphatase activity, ATPase activity, p53 binding, repressing transcription factor binding, fatty-acyl-CoA binding, and glucocorticoid receptor binding were the most highly enriched terms (Figure 7C). All mRNAs annotated involved in these GO terms were listed in Table S6. Here, the majority of mRNAs appear to be associated with metabolic process, cellular senescence, and mitochondria. For instance, ATP binding and ATPase activity are closely related to energy metabolism and mitochondrial function (42). Mitochondria produce ATP and are a source of ROS with potentially toxicity (43). Aging is characterized by gradual mitochondrial dysfunction and increased risks for metabolic disorders (44). Together, this GO analysis suggests that BAT dysfunction has a close relationship with mitochondria disorders with aging.

Furthermore, the KEGG results indicated that DE lncRNAs were involved in ubiquitination-mediated proteolysis, DNA replication, microRNAs in cancer, sphingolipid signaling pathway, lysine degradation, ribosome biogenesis in eukaryotes, cell cycle, metabolic pathways, and NF-kappa B and p53 signaling pathways (Figure 7D). All mRNAs annotated involved in these pathways were listed in Table S7. Then, we used these pathways to construct a pathway network to investigate the key pathways in brown fat dysfunction with aging. As is shown in Figure 7E, the exchanges with these pathways mainly depended on the existence of cell cycle and p53 signaling pathway. Cell cycle and senescence machinery may regulate brown adipocyte formation (45, 46). As a crucial regulator of cell cycle, p53 has an impact on the maintenance of adipocytes function and the regulation of energy metabolism and homeostasis (45). Thus, these two pathways may have the function in BAT dysfunction with aging, consistent with previous work (Figure 6C).

### NONMMUT024512 and n281160 may Involve in Regulation of Pparα Expression

Previous studies reveal that lncRNA could affect the transcription of their neighboring coding genes (47). In the present study, we determined that the expression of NONMMUT024512 and n281160 was significantly downregulated in BAT from old mice. Intriguingly, their neighboring coding gene Pparα is well-known as a key brown thermogenesis regulator. We further applied the Integrative Genomics Viewer (IGV) to show genome track visualization with reads density information available for these two lncRNAs in Figure S3A. There were a few alignments with reads mismatching the reference genome, as indicated by color. However, many more alignments consistent with the reference were represented in light gray. Moreover, we used qPCR to verify the correlation between Pparα and these two lncRNAs in BAT. The results revealed that NONMMUT024512 was correlated with Pparα (*r* = 0.7055, *p* < 0.0001), and n281160 had the stronger correlation with Pparα (*r* = 0.8303, *p* < 0.0001; Figure S3B). We further performed the correlation analyses between 4 randomly selected lncRNAs (CUFF.134482.1, NONMMUT058471, TID_00007994, and n287087) and Pparα. Our result indicated that the correlation between these lncRNAs and Pparα was poor (Figure S3C).

**Figure 8:**
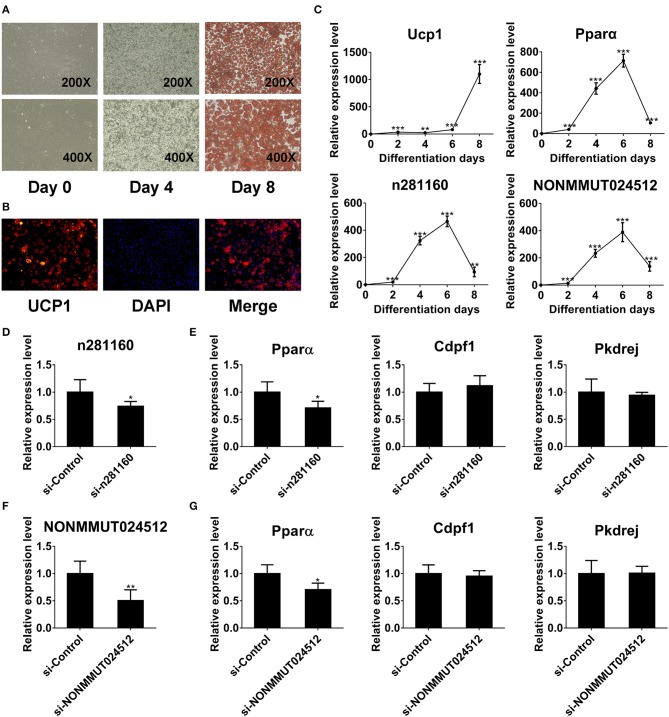
NONMMUT024512 and n281160 may involve in regulation of Pparα expression. **(A)** Representative microscopic images at day 0 and 4 of differentiation, and Oil red O staining at day 8. **(B)** UCP-1 immunofluorescence of primary brown adipocytes at day 8 of differentiation (original magnification, ×200). **(C)** Transcriptional levels of Ucp1, Pparα and two candidate lncRNAs at indicated times during the differentiation of brown adipocytes. **(D–G)** The expression of Pparα in adipocytes transfected with siRNAs targeting NONMMUT024512 or n281160 at day 6 of differentiation. Data are presented as the mean ± standard deviation (S.D.). Statistical significance evaluated by unpaired Student's *t*-test is reported as *p < 0.05, **p < 0.01, and ***p < 0.001.

To investigate the expression of two candidate lncRNAs in the differentiation process of brown adipocytes, we detected their transcriptional level at indicated times during differentiation. First, the degree of lipid accumulation was evaluated with light microscopy and Oil red O staining. The formation of lipid droplets gradually increased during differentiation. At day 8 of induction, brown adipocytes were filled with large and abundant lipid droplets (Figure 8A). We also conducted immunofluorescence against UCP1 to identify these cells as brown adipocytes. As expected, brown thermogenic marker UCP1 was enriched in differentiated cells (Figure 8B). Furthermore, we detected the expression of Ucp1 in the differentiation process of brown pre-adipocytes. Ucp1 expression was gradually induced during differentiation (Figure 8C). These results demonstrated that pre-adipocytes from mouse interscapular BAT had been differentiated into classic brown adipocytes. On this basis, we further examined the expression of two candidate lncRNAs during differentiation. Interestingly, the expression of these two lncRNAs was gradually induced during differentiation and peaked at day 6, consistent with Pparα expression (Figure 8C).

Moreover, to evaluate the impact of NONMMUT024512 and n281160 on Pparα expression, we performed RNAi-knockdown experiments using siRNAs though transfecting them into primary pre-adipocyte cultures, followed by induction of differentiation. Considering the expression trend of Pparα during differentiation (Figure 8C), we harvested cells with Trizol at day 6 of induction. Then, we observed that the knockdown of these two lncRNAs resulted in the reduction of Pparα expression with no impact on the expression of other genes in the vicinity or upstream, such as Cdpf1 and Pkdrej (Figures 8D–G). These two lncRNAs seem to be involved in BAT dysfunction by affecting Pparα expression *in cis*.

## Discussion

BAT is able to rapidly generate abundant heat through activating UCP1 (48). This UCP1-driven thermogenesis has been shown to decrease circulating lipids and glucose in rodents and induce energy expenditure in humans (49). Emerging evidence suggests that thermogenic fat significantly influences whole body metabolism in humans (50). However, in both rodents and humans, brown fat mass and thermogenic activity diminish with aging (51), thereby contributing to increased fat accumulation and the enhanced incidence of metabolic disorders, such as diabetes (52). A previous study revealed that BAT is progressively populated by white-like morphological adipocytes in aging, and older mice are unable to mobilize intracellular fuel reserves from BAT (24). Here, we also found morphological and molecular abnormalities of BAT in two different age groups. We observed that multilocular brown adipocytes markedly disappeared in older mice. In contrast, the lipids stored in brown adipocytes are large and unilocular, indicating a decrease in lipolytic capacity. Consistent with these findings, UCP1 protein expression was strikingly diminished in old animals, signifying the impairment in thermogenic activity. We also observed significant reductions in the mRNA level of Ucp1 and Pgc1α in BAT. Furthermore, we observed alterations in senescence-associated gene expression. The p16^Ink4a^ protein serves as a cell cycle inhibitor in cellular senescence (53). By activating the senescence pathway, p16^Ink4a^ induces preadipocyte senescence and blocks cold-induced beiging (54). A recent animal study (55) revealed that cellular senescence in adipose tissue was accompanied by the upregulation of p16^Ink4a^. Additionally, Klotho, an important co-receptor of fibroblast growth factor 21 (56), plays a role in systemic glucose metabolism and adipocyte differentiation (57) and has been shown to be indispensable for cold-induced beige fat thermogenesis (58). The serum levels of Klotho are reduced with aging, and Klotho-deficient mice exhibit short lifespans and aging characteristics (59). In the present study, we observed a marked increase in p16^Ink4a^ expression and a dramatic reduction in Klotho expression in BAT with aging, as described previously (33, 55). These findings, along with alterations in BAT morphology and thermogenic gene expression, suggest that brown thermogenic activity may be vestigial with aging.

Cold exposure can stimulate energy formation through brown thermogenic activity and induce metabolism to enhance energy expenditure (60). However, cellular senescence in precursors of brown adipocytes may interfere with cold-induced brown adipogenesis in older mammals. Furthermore, the molecular mechanism of this age-related dysfunction in BAT hasn't been identified. Present studies have revealed a crucial impact of lncRNAs on brown fat development and differentiation. lncRNA H19 was negatively related to body mass index in humans and was particularly essential for brown adipocytes and energy metabolism. This lncRNA promoted brown adipogenesis and thermogenesis *in vitro* and ensured energy dissipation *in vivo* (61). Another recent study using a knockout strategy revealed that lnc-dPrdm16, a lncRNA located divergently from Prdm16, was critical for brown adipocyte differentiation and maintaining a BAT-selective gene programme in mature adipocytes (62). Despite these advancements, the lack of an integrated lncRNA catalog in BAT with aging has hampered the understanding of lncRNA in less functional BAT. Therefore, before the regulatory roles of lncRNAs in thermogenic dysfunction can be assessed, establishing an integrated brown adipose lncRNA catalog is required.

Here, we performed RNA-seq on young and old mice to examine dynamic changes in mRNA and lncRNA expression in BAT with aging. Altogether, 3237 mRNAs and 1312 lncRNAs were identified as differentially expressed between the two groups (FC ≥ 2.0 and FDR ≤ 0.05). Moreover, the expression of a few lncRNAs and mRNAs validated by qPCR was analogous to that in the sequencing data. Furthermore, STRING and k-score analyses indicated that the core genes in the protein interaction network were enriched in cell cycle and p53 signaling pathway. GSEA also revealed that the genes in BAT of old mice may not only regulate lipid and glucose metabolism but also participate in brown fat dysfunction.

In the same way, GO and pathway analyses were performed to identify the potential functions of DE lncRNAs using their *cis*- and *trans*-regulated coding genes. GO analysis demonstrated that DE lncRNAs were highly associated with cellular response to stress, cellular response to DNA damage stimulus, regulation of cellular senescence and mitochondria. Cellular senescence is an irreversible growth arrest in response to various cellular stresses, DNA damage and mitochondrial dysfunction (63, 64). Oxidative stress induces DNA damage and ROS production (65), leading to cellular senescence characterized by mitochondrial dysfunction (66). BAT has numerous mitochondria and achieves brown thermogenesis via activating UCP1 located within the inner mitochondrial membrane (48). Thus, mitochondrial dysfunction may cause the diminished thermogenic function of BAT. Moreover, pathway analysis revealed significant changes in “cell cycle,” “metabolic pathways,” and “p53 signaling pathway” in BAT. Metabolic pathways are relevant to nutritional sensing and converge with mechanisms involving crucial regulators of energy homeostasis (67). In addition, aging is a primary factor risk for metabolic diseases, such as obesity and diabetes (52, 68), suggesting the correlation between age-related signaling and metabolic pathways. In adipose tissue, activating p53 signaling pathway could contribute to insulin resistance related to obesity and diabetes (69). Together, these altered lncRNAs are associated with metabolic and age-related signaling pathways along with various biological processes involved in mitochondrial dysfunction, and may play important regulatory roles.

The transcription of non-coding genes has been identified to affect the expression of nearby protein-coding genes through RNA-protein interactions (47). Lnc-PCTST is a potential tumor suppressor that suppresses pancreatic cancer progression by downregulating transforming acidic coiled-coil 3, which is located ~170 kb downstream from lnc-PCTST (70). Another lncRNA, HOTTIP, transcribed from the 5′ end of the HOXA locus, was reported to coordinate the activation of multiple 5′HOXA genes *in vivo* (71). Based on preliminary results, we focused on two candidate lncRNAs, NONMMUT024512 and n281160, which are both located around Pparα *in cis*. Furthermore, we also found that the expression of these two lncRNAs was gradually induced during differentiation and reached a peak at day 6 of differentiation, consistent with Pparα expression. Given that lncRNAs can *in cis* regulate genes that are positioned in the vicinity of their transcription sites in nuclei, so we supposed that these two lncRNAs may affect the expression of their adjacent gene Pparα. Thus, we further adopted loss-of-function strategy by siRNA to unmask the impact of these two lncRNAs on Pparα expression. We observed that the expression of Pparα was decreased in adipocytes transfected with siRNAs targeting these two lncRNAs at day 6 of differentiation. These findings partially indicated the regulatory relationship between these two lncRNAs and Pparα. Pparα is the important transcriptional factor that coordinates the activation of thermogenesis and lipolysis in brown fat (72). Abundant expression of Pparα differentiates brown fat from white (73). Increasing evidence supports the relation of PPARα and metabolic diseases, including obesity, dyslipidemia, fatty liver and diabetes (74). Taken together, these two lncRNAs appear to play a role in brown thermogenic activity through their neighboring gene Pparα *in cis*.

In the present study, we profiled DE mRNAs and lncRNAs in BAT of young and old mice. Based on previous results, we focused on two lncRNAs (NONMMUT024512 and n281160), which appear to play a role in diminished brown thermogenesis via Pparα *in cis*. However, further investigation of the molecular and biological functions of these lncRNAs in BAT with aging is required.

## Data Availability Statement

All datasets generated for this study are included in the article/Supplementary Material.

## Ethics Statement

All animal experiments were approved by the Nanjing Medical University Committee on Care and Use of Animals, and conducted in conformity with the Guide for the Care and Use of Laboratory Animals published by the U.S. National Institutes of Health (NIH Publication number 85–23, revised 1996).

## Author Contributions

XC and TZ designed the study. JF and HX wrote the manuscript. FP, JH, YW, NL, XZ, CJ, YH, HZ, and LY performed the experiments and analyzed the data. All authors reviewed and edited the manuscript.

### Conflict of Interest

The authors declare that the research was conducted in the absence of any commercial or financial relationships that could be construed as a potential conflict of interest.
