# Supplementary material for: An Integrated Analysis of mRNA and lncRNA Expression Profiles Indicates Their Potential Contribution to Brown Fat Dysfunction With Aging
Source: Front Endocrinol (Lausanne). 2020 Feb 17;11:46. doi: 10.3389/fendo.2020.00046 (PMC7039067; doi:10.3389/fendo.2020.00046)
Supplement: Supplementary file 8 [file Data_Sheet_1.PDF]

Supplemental Figures

An integrated analysis of mRNA and lncRNA expression profiles indicates their potential contribution to brown fat dysfunction with aging

Jie Feng[1, 2, +], Haoqin Xu[2, +], Fenghui Pan[3], Jiaojiao Hu[1], Yulin Wu[2], Ning Lin[2], Xiaoxiao Zhang[1], Chenbo Ji[1], Yun Hu[3], Hong Zhong[1], Linping Yan[1], Tianying Zhong[1, *] & Xianwei Cui[1, *]

[1]*Nanjing Maternity and Child Health Care Hospital, Women's Hospital of Nanjing Medical University, Nanjing, Jiangsu 210004, China.*

[2]*Jiangsu Institute of Planned Parenthood Research, Nanjing, Jiangsu 210036, China.*

[3]*Department of Geriatrics, Drum Tower Hospital Affiliated to Nanjing University Medical School, Nanjing, Jiangsu 210008, China.*

[+]**These authors contributed equally to this work**

[*]**To whom correspondence should be addressed:** Nanjing Maternity and Child Health Care Hospital, Women's Hospital of Nanjing Medical University, Nanjing, Jiangsu 210004, China. Phone: 86-25-52226159, Fax: 86-25-52226159. E-mail: xwcui@njmu.edu.cn (X.W. Cui); E-mail: zhongtianying@hotmail.com (T.Y. Zhong).

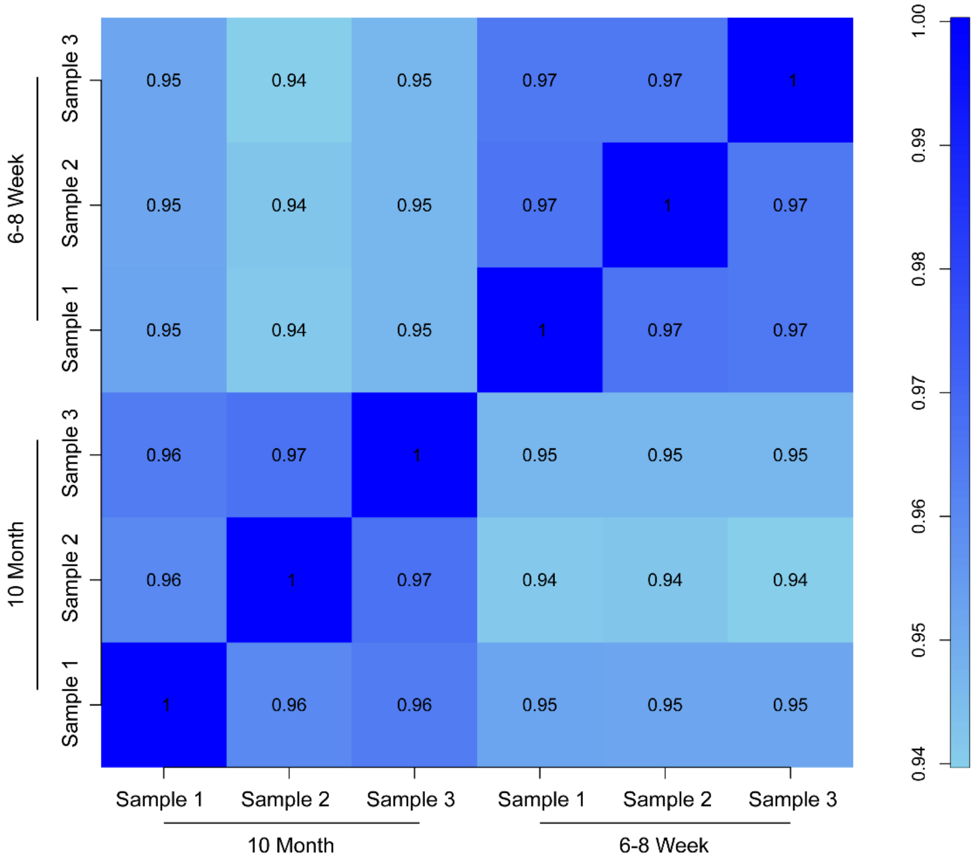

FIGURE S1 | Correlation analysis of BAT samples between 6- to 8-week-old and 10-month-old mice.

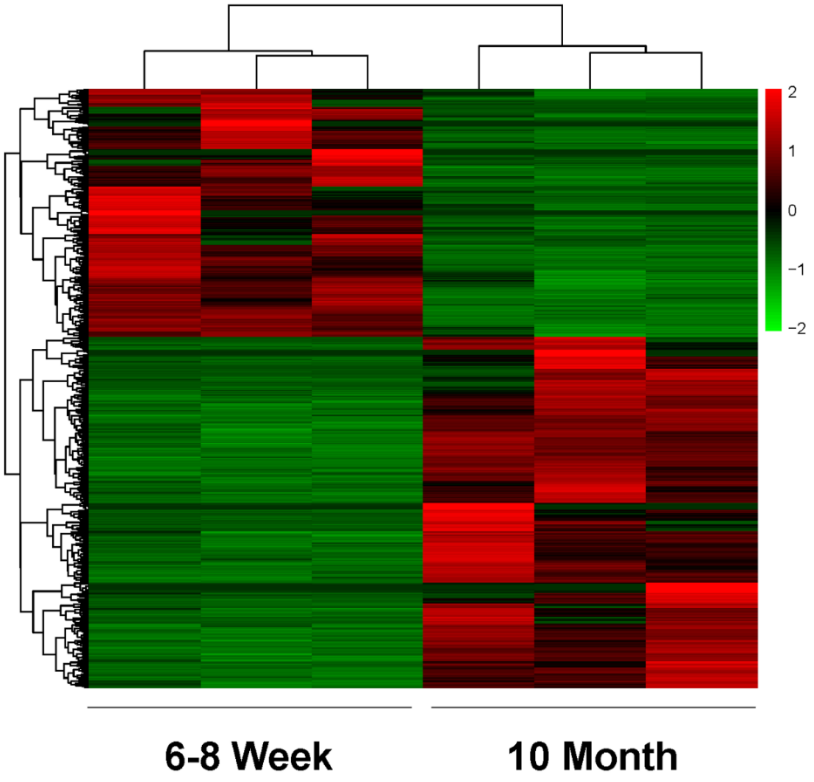

FIGURE S2 | Clustered heat map analysis of differentially expressed lncRNAs between young and old groups.

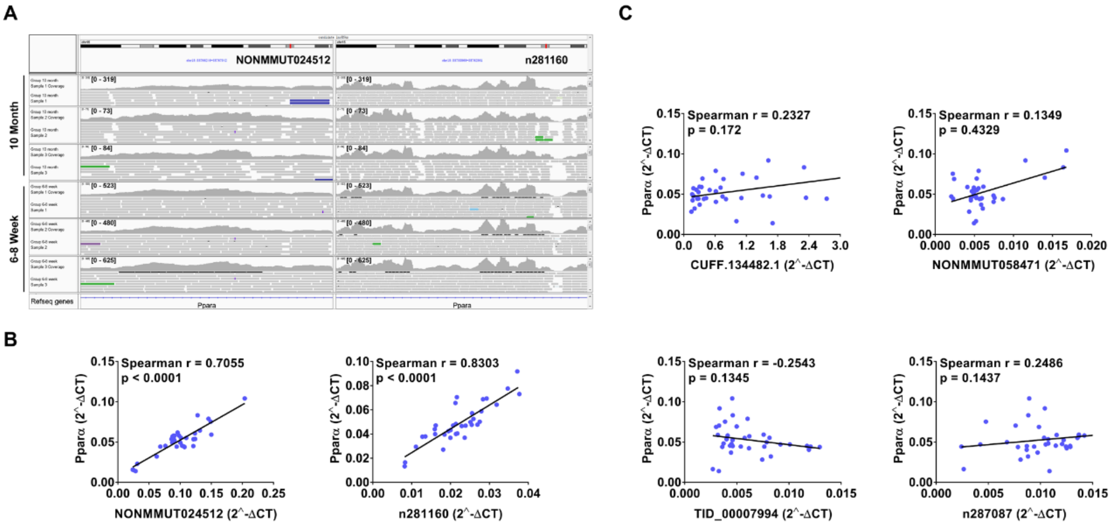

FIGURE S3 | NONMMUT024512 and n281160 may involve in regulation of Pparα expression. **(A)** View of read alignments from BAT samples, displaying regions of NONMMUT024512 and n281160. Each panel includes tracks for coverage plot and read alignments. Alignments are represented in light gray with reads mismatching the reference indicated by color. **(B)** The correlation of these two lncRNAs and Pparα was analysed by Spearman coefficients. **(C)** Correlation analysis between the expression of 4 randomly selected lncRNAs and Pparα in BAT samples from 6- to 8-week-old and 10-month-old mice.
